# Effect of Ultrasound-Assisted, Microwave-Assisted and Ultrasound-Microwave-Assisted Extraction on Pectin Extraction from Industrial Tomato Waste

**DOI:** 10.3390/molecules27041157

**Published:** 2022-02-09

**Authors:** Patareeya Lasunon, Nipaporn Sengkhamparn

**Affiliations:** Faculty of Interdisciplinary Studies, Nong Khai Campus, Khon Kaen University, Nong Khai 43000, Thailand; lpatar@kku.ac.th

**Keywords:** ultrasound-assisted extraction, microwave-assisted extraction, ultrasound-microwave-assisted extraction, fuzzy analytical method, Pectin

## Abstract

This work aimed to study the effect of ultrasound-assisted (UAE), microwave-assisted (MAE), and ultrasound-microwave-assisted (UMAE) methods for pectin extraction from industrial tomato waste. The overall performance index from the fuzzy analytical method with three criteria, pectin yield, galacturonic acid, and lycopene content, was applied to evaluate the best extraction conditions by using the weight of 75, 20, and 5, respectively. The UAE conditions was performed at a temperature of 80 °C for 20 min with the variations in the extraction pH and the solid liquid (SL) ratio. The best UAE conditions with high pectin yield, and high total carboxyl group, as well as a lycopene content, was the pH of 1.5 and the SL ratio of 1:30. The MAE conditions was performed with variations in the microwave powers and times. The results showed that the best MAE conditions were 300 W for 10 min, which gave high pectin yield with high galacturonic acid and lycopene content. Various conditions of UMAE at the best conditions of MAE and UAE were performed and exhibited that the UAE had more positively affected the pectin yield. However, the FTIR spectra of obtained pectins from different extraction techniques showed a similar pectin structure.

## 1. Introduction

Tomato fruits (*Solanum lycopersicum* L.) are healthy plants, which can be consumed as a fresh vegetable or can be processed and made into many food products, such as ketchup, paste, sauce, and juice [1]. A tomato which cultivated in the world has been used for produce tomato food products for almost 40 million tons in 2021 [2]. Tomato waste from commercial tomato manufacturing has been used as raw material for the extraction of bioactive compounds, such as lycopene extraction [3,4], phenolic extraction [1], and pectin extraction [5,6,7]. Grassino et al. [6] extracted pectin from tomato peels by reflux at 90 °C for 24 and 12 h and ammonium oxalate/oxalic acid used as solvent. 

Pectin is used as a functional ingredient in the food industry (for example, as a gelling agent, a thickening agent, and as a stabilizer). The backbone of pectin is composed of α-(1,4)-D-galacturonic acid on which some of galacturonic acid can be methyl-esterified thus, impacting its functional properties [8]. Traditionally, pectin can be extracted by hot mineral acids such as sulfuric, nitric, phosphoric, or hydrochloric acid at pH 1.3–3.0 and the temperature of 60 and 100 °C for 1 h [9,10]. Citric acid is an organic acid with a chelating property and has been studied for use as a solvent for pectin extraction, such as sugar beets [11], mango peels [12,13]. It was reported that the citric acid had given a higher pectin yield than mineral acid [7,12]. Many techniques have been developed for pectin extraction, such as microwave heating, ultrasonication, and a super high-frequency electromagnetic field [14].

Ultrasound-assisted extraction (UAE) is an extraction technique which uses sound waves at frequencies higher than 20 kHz [15,16]. The UAE has been used for bioactive compound extraction due to its shorter period, higher yield, lower energy, and solvent consumptions. Many research studies have been conducted on UAE for pectin extraction from many plants, such as mango peels [13], jackfruit peels [17], and tomato waste from the canning industry [5] and from the tomato paste industry [7]. Grassino et al. [5] extracted pectin from tomato wastes from the canning industry by using ultrasound techniques at the extraction temperatures of 60 °C and 80 °C with ammonium oxalate/oxalic acid, in which these conditions gave a comparable yield, but took a shorter time. Sengkhamparn et al. [7] reported that the appropriate UAE condition for pectin extraction from tomato waste, derived from the tomato paste industry, was a temperature of 80 °C and a time of 20 min with citric acid at a pH of 2.0. In this work, the pectin yield was slightly low. However, other factors, such as pH and also the solid/liquid ratio, had affected the pectin yield. Moorthy et al. [17] extracted pectin from the wastes of jack fruit peels by UAE and reported that an increase in the SL ratio from 1:10 to 1:18 mL/g had increased the pectin yield. Moreover, they suggested that when a lower pH is used, a higher pectin yield is gained. 

Microwave-assisted extraction (MAE) is another type of extraction technique which uses a non-ionizing wave at frequencies ranging from 300 MHz up to 300 GHz [18,19,20]. These waves can generate rapid heat, inducing cellular damage and enhancing the solvent diffusion, which causes the interesting compound to transfer to the solvent medium [21,22]. Tongkham et al. [23] reported that with dragon fruit peel, a microwave power of 450 W for 5 min had given a high pectin yield with less degradation. Moreover, Rodsamran and Sothornvit [24] compared the solvent for extracting pectin from lime peel by MAE and found that the citric acid had caused a higher quality of pectin in which the pectin content had been found to have a higher equivalent weight and a higher degree of esterification as compared to other conventional extraction processes. Dranca et al. [25] reported that the pectin yield from *Malus domestica* ‘Fălticeni’ apple pomace could be better improved by using MAE rather than by UAE. 

Moreover, a combination of UAE and MAE has been used for pectin extraction. Bagherian et al. [26] reported that the grapefruit pectin yield from MAE was comparable to that from conventional, which required 90 min to perform. When comparing the UAE to the conventional method, the UAE had given a three times higher extraction rate. Moreover, they also stated that the UAE followed by MAE (UMAE) had given an even greater yield than the others. Moreover, Liew et al. [27] studied the optimum pectin extraction conditions from citrus fruit peels and found that the UAE at a pH of 1.80 and radiation for 27.52 min, followed by MAE at 643.44 W for 6.40 min, had provided a higher pectin yield (38.0%) than the MAE followed by UAE, as well as a pectin yield that was higher than MAE and UAE, respectively. However, there have been no research studies comparing the effects of the UAE, MAE, and the different UMAE conditions by using citric acid for pectin extraction from industrial tomato waste, as well as by comparing the lycopene content in the obtained pectin.

The fuzzy analytical method (FAM) is a mathematical technique for making a decision by generating the results (assessment scores) into a fuzzy scale (0 to 10) and then integrating the fuzzy grade matrix with their relative weights into the overall index. This index number can be used for comparison and decision the suitable conditions. FAM is an evaluation tool for assessment systems in food product application such as for food product quality control [28], for sensory evaluation [29,30,31], and for decision of optimum extraction conditions [23,32].

Therefore, this study aimed at examining the UAE, MAE, and the various conditions of UAE, which was then followed by MAE in order to extract pectin from tomato wastes. Moreover, the chemical properties of the obtained pectin have been also determined. 

## 2. Results

### 2.1. Ultrasound-Assisted Extraction (UAE)

**Figure 1:**
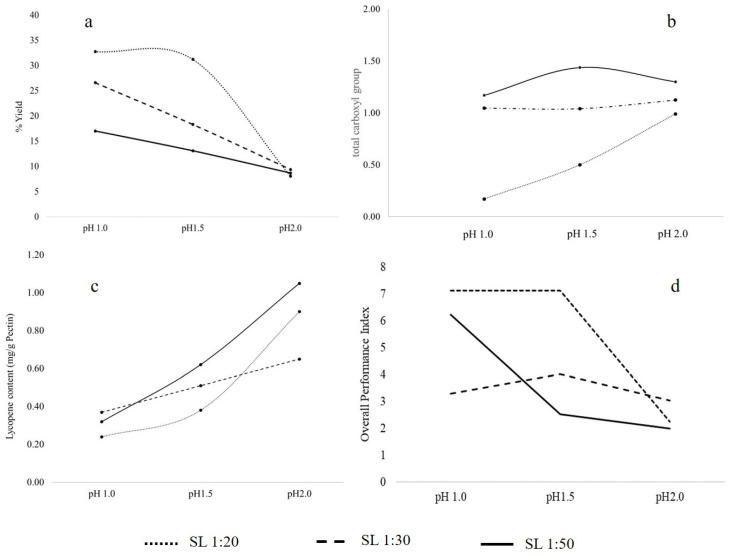
The pectin yield (**a**), total carboxyl group (**b**) and lycopene content (**c**), and overall performance index (**d**) of each condition.

The pH values (1.0, 1.5, and 2.0) and the SL ratios (1:20, 1:30, and 1:50) of the UAE conditions were studied. The obtained pectin yield is shown in Figure 1a. The results showed that the extraction at the lower pH values and the lower Solid Liquid (SL) ratios had increased the pectin yield. The increase of pH values from 1.5 to 2.0 the pectin yield was decreased about 74.14, 48.75, and 50.74% for the SL ratio of 1:20, 1:30, and 1:50, respectively. Besides, with the increase of SL ratio from 1:20 to 1:50 the pectin yield has been decreased about 18.81 and 58.04% for the pH of 1.0 and 1.5, respectively. Moreover, the UAE conditions with pH of 1.0 and 1.5 and SL ratio of 1:20 were significantly comparable and had given the highest pectin yields (32.77% and 31.23%, respectively). 

The obtained pectin form each UAE condition was simply characterized. The total carboxyl groups of obtained pectin from each condition were determined using FT-IR spectroscopy. The results (Figure 1b) showed the total that the carboxyl group was in the range of 0.17–1.43 and it found that the lower pH and SL ratio had gained the lower total carboxyl group. Besides, the trapped lycopene content in the pectin was determined. The results (Figure 1c) showed that the lycopene content was found to be high at a pH value of 1.5, which was higher than the pH values of 1.0 and 2.0. Based on a decrease in pH from 2.0 to 1.5, the amount of trapped lycopene increased.

The best UAE conditions have been determined using the fuzzy analytical method with three criteria (pectin yield, total carboxyl group, and lycopene). The weight of each criterion for calculating the overall performance index from fuzzy grade was 70, 25, and 5 for pectin yield, total carboxyl group, and lycopene, respectively. The results showed that the extraction at pH of 1.5 and the SL ratio of 1:20 were the best conditions which contained the highest overall performance index (7.12). These conditions could be used for further studies.

### 2.2. Microwave-Assisted Extraction (MAE)

In this study, different MAE conditions (the microwave powers of 300 W, 450 W, and 600 W and extraction times of 3, 5, and 10 min) were performed. The pectin yield from each condition is shown in Table 1. The results exhibited that at the same extraction time, the pectin yield was increased when a higher microwave power was supplied. However, it was found that when higher microwave powers (600 W) were used for a longer time, there was a slight decrease in the pectin yields. At the extraction time of 3 min, the pectin yield was increased from 9.43% (300 W) to 12.57% and 22.85% when extraction at microwave power of 450 W and 600 W, respectively. On the other hand, the obtained pectin yield was decreased from 29.67% at the extraction time of 5 min to 28.28% at that of 10 min. 

Moreover, that microwave power and extraction time also had affected on galacturonic acid (GalA) content. The results (Table 1) showed that when the higher microwave power was used and the longer extraction was performed, the pectin had provided higher GalA contents. The lycopene content had been impacted by the extraction time rather than by the microwave power. Higher amounts of lycopene in the pectin were derived from using a shorter extraction time. 

The overall performance index of each condition was calculated regarding fuzzy analytical method. In this work, the three criteria were pectin yield, galacturonic acid and lycopene content, and the weight of each criterion was 75, 20, and 5, respectively. The overall performance index of each condition is shown in Table 1. The results exhibited that the great MAE conditions at 300 W for 10 min had the highest overall performance Index (7.23). These MAE conditions have been used for pectin extraction with UMAE and is discussed in the next part.

### 2.3. Ultrasound-Microwave-Assisted Extraction (UMAE)

The UMAE was carried out under four different conditions as follows: (1) the best condition of UAE (at the temperature of 80 °C for 20 min, a pH value of 1.5, and the SL ratio of 1:20) followed by the best conditions of MAE (microwave power of 300 W for 10 min) [UAE + MAE], (2) the best conditions of UAE followed by half the time duration of MAE (300 W for 5 min) [UAE + 1/2 MAE], (3) half the time duration of UAE (80 °C for 10 min) followed by the best conditions of MAE [1/2 UAE + MAE], and (4) half the time of UAE followed by half the time duration of MAE [1/2 UAE + 1/2 MAE]. The pectin yield from each condition was compared to the UAE and MAE conditions. The results are shown in Table 2.

The results (Table 2) showed that the pectin yield from the MAE conditions (28.25%) had been slightly higher than the pectin yield from the UAE conditions (30.12%). The pectin yield was increased from 28.25% or 30.12 to 34.16% when the combination of UAE followed by MAE was performed. In addition, even though the UMAE conditions with the extraction time of MAE had been reduced twice, the pectin yield was comparable to the full extraction of MAE. Inversely, with the reduction of the UAE time, the pectin yield decreased from 34.39% to 19.68% and 26.49% when using half the time of MAE and the full time of MAE, respectively. 

Considering GalA content, it was found that the UAE pectin (13.41 µg/L) had shown the lower GalA content the MAE pectin (16.86 µg/L). The GalA content of obtained pectin was positively related to pectin yield which the higher pectin yield was obtained, the higher GalA was found. For trapped lycopene content (Table 2), it was found that the MAE pectin (27.05 µg/g Pectin) had contained higher trapped lycopene than UAE pectin (9.99 µg/g Pectin). The lycopene was increased from 27.05 µg/g Pectin when supping MAE to about 8.53–26.85 µg/g Pectin when the combination of UAE followed by MAE has been performed. 

The fuzzy analytical method has been applied in order to evaluate the best extraction conditions by using 3 criteria and weight as same as MAE. The results (Table 2) showed that the extraction with UAE followed by half of MAE revealed the highest overall performance index (8.99).

**Figure 2:**
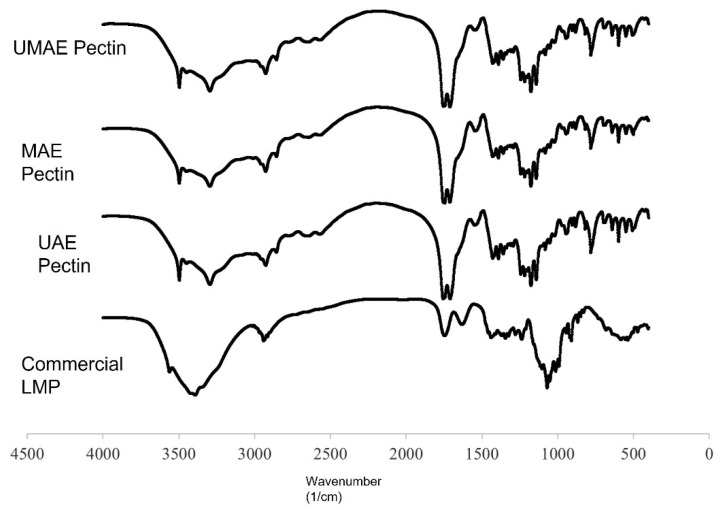
FT-IR spectra of UAE, MAE, and UMAE pectin from tomato waste and commercial pectin.

The structure of the obtained pectin from UAE, MAE, and UMAE was characterized and compared to commercial pectin (Figure 2). The FTIR results can be pointed that the extracted was pectin and the extraction techniques were not affected by the pectin structure.

## 3. Discussion

### 3.1. Ultrasound-Assisted Extraction (UAE)

Previously, pectin has been successfully extracted from industrial tomato waste by UAE at 80 °C for 20 min with citric acid at a pH of 2.5, which gave the highest yield (10%) and this yield was higher than any other mineral acid, hydrochloric acid, or nitric acid had given [7]. However, this yield was still lower. Therefore, to improve the pectin yield, the pH values, and the solid-liquid (SL) ratios of the UAE conditions were studied. 

The results (Figure 1a) showed that the decrease in pH values and SL ratio increased the pectin yield. This can be explained by the fact that the high acidity would destroy the cell wall of the raw material and thus would allow the solvent to penetrate and dissolve the pectin into the extracting solvent. This effect of low pH was also found in the UAE of pectin obtained from jackfruit [17] and pomegranate peel [33]. On the other hand, the higher SL ratio resulted in the decrease of pectin yield this was probably due to the decrease of the power density of ultrasound waves. This result was in accordance with the research conducted by Moorthy et al. [17]. Moreover, the highest pectin yields were found in UAE conditions with pH of 1.0 and 1.5 and SL ratio of 1:20 (32.77% and 31.23%, respectively). This yield was comparable to findings from a study by Grassino et al. [6] (32.6%) in which pectin was extracted from tomato peels by using ammonium oxalate/oxalic acid under reflux at 90 °C for 24 and 12 h. However, it was lower than in the study by Grassino et al. [5] in which pectin had been extracted from tomato waste using a two-step process of UAE at 80 °C, however, in this study, the conditions were performed for a shorter time. Moreover, these conditions had provided a higher yield than pectin from jackfruit peel (14.5%), which had been extracted by UAE at 60 °C for 24 min with a pH value of 1.6 and SL ratio of 1:15 [17]; and higher than pectin from grapefruit peel (27.34%), which had been extracted by UAE at 66.71 °C for 27.95 min with a pH value of 1.5 and SL ratio of 1:50 [34]. These results were due to many experimental factors: the raw materials themselves, the extracting solvents, and the UAE conditions. However, the best UAE conditions were an extraction pH of 1.5 and an SL ratio of 1:20 or 1:30. Moreover, within these conditions, the pectin yield had improved by approximately 20% as compared to our previous work [7].

FT-IR spectroscopy was one of techniques that was reported that can be used to determine the total carboxyl groups of pectin [27]. The results (Figure 1b) showed that the decrease of pH caused the lower total carboxyl group of obtained pectin. Moreover, the conditions of high-density sound waves, which was performed at a low SL ratio, had also gained a low total number of carboxyl groups. This could point to the heavy conditions, high acidity, and dense of sound waves, which could have degraded the pectin structure. 

The color of the obtained pectin was reddish-yellow, this color was probably due to the lycopene content in pectin. Jazaeri et al. [35] reported that pectin can form a hydrocolloidal substance with lycopene during processing. Therefore, the trapped lycopene content in the pectin was determined. The results (Figure 1c) showed a decrease in pH from 2.0 to 1.5, the amount of trapped lycopene increased, which was probably due to more disruption in the cell walls caused by higher acidity and hence the increase of the lycopene dissolved. However, with a decrease in the pH from 1.5 to 1.0, the trapped lycopene was decreased, which may be due to the structure of obtained pectin. 

To evaluate the best MAE conditions, the fuzzy analytical method (FAM) was applied. This method can evaluate the best conditions by using more than one criterion. The FAM calculated the overall performance Index by using 3 criteria which were pectin yield, lycopene and galacturonic acid content. Each criterion of each condition was transferred to fuzzy (performance) score in the same score range of 0–10. After that, the performance score of each criterion was calculated to each fuzzy grade and then it was concluded to overall performance Index with each wight. This due to this work aim to extract pectin with high pectin yield. Therefore, the weight of each criterion was 75, 20, and 5 for pectin yield, galacturonic acid content, and lycopene content, respectively. The highest overall performance index of UAE conditions was the UAE conditions at a pH of 1.5 and SL ratio of 1:20. These conditions would obtain the high pectin yield and high GalA, as well as lycopene. Therefore, the UAE condition at a pH of 1.5 and SL ratio of 1:30 had given a high yield of pectin and total carboxyl groups, as well as low lycopene. The same extraction pH values and SL ratio of the UAE from this part of the study were further used for the MAE condition in the next section.

### 3.2. Microwave-Assisted Extraction (MAE)

The same extraction pH value and SL ratio of the UAE were further used for the MAE condition. In MAE conditions with the microwave powers of 300 W, 450 W, and 600 W and extraction times of 3, 5, and 10 min were performed. The pectin yield from each condition revealed that the microwave power has positively affected the pectin yield. It could be explained that the microwave energy had generated heat when higher powers were used, and therefore higher heat was generated. The generated heat could disrupt the cell wall and thereby transfer the compound to the extracting solvent. This result was in agreement with Dranca et al. [36] who reported that the higher microwave power, the higher pectin yield. However, the higher microwave powers (600 W) were used for a longer time, the pectin yields slightly decreased. This result was also found by Thirugnanasambandham et al. [37] in which when the microwave power was higher than 400 W, the pectin yield from dragon fruit peel was decreased. In this study, the extraction at the microwave power of 300 W for 10 min had provided the highest pectin yield. Maran et al. [38] reported that the optimum microwave power for pectin extraction from the waste of *Citrullus lanatus* fruit rinds was 477 W for 128 s, while Tongkham et al. [23] suggested that a microwave power of 450 W for 5 min at a pH of 2.0 and with an SL ratio of 1:30 could offer a better pectin yield with high pectin molecules. Moreover, Thu Dao et al. [39] reported the highest pectin yield from passion fruit peel can be extracted using MAE with microwave power of 218 W for 12 min at pH of 2.9 and SL ratio of 1: 57. This was probably due to the other extraction factors, such as the extraction pH and the SL ratio as well as an extraction solvent. 

Considering GalA and lycopene content. The results (Table 1) showed that the higher microwave power and longer extraction time exhibited the higher GalA contents. This was in agreement with the higher pectin yield. This result was in agreement with Sucheta et al. [40] who stated that the higher pectin yield may cause higher GalA in pectin from black carrot. On the other hand, the trapped lycopene content in the pectin was determined and the results showed that longer extraction time was negatively related to the lycopene content. However, no research studies have reported the structure of the trapped lycopene and pectin. 

To evaluate the best MAE conditions, the fuzzy analytical method (FAM) was applied with the weight of criteria as same as for evaluating the best UAE (75:20:5 for pectin yield, galacturonic acid, and lycopene content, respectively). The highest overall performance index of MAE conditions was the extraction at a microwave power of 300 W for 10 min which had yielded the high GalA content and had also given the highest pectin yield, but the lycopene content had remained low. These MAE conditions have been used for a further pectin extraction with UMAE.

### 3.3. Ultrasound-Microwave-Assisted Extraction (UMAE)

The UAE followed by MAE (UMAE) is an efficient extraction method and gives a higher pectin yield than when performed by only one method, as well as the MAE followed by UAE [27], therefore, the different conditions of UMAE were performed and were compared to UAE and MAE. The UMAE was carried out under 4 different conditions in order to eliminate 2 extracted factors, pH and Solid Liquid ratio. The pectin yield from each condition (Table 2) showed that when comparing the MAE and UAE, the MAE showed more efficiency than UAE in which higher pectin yield and also shorter time consuming. In addition, each extraction technique has vaguely different mechanisms. MAE technique was the heating the food matrix with electric field generates resulted in the vibrations of polar water molecules, enhanced of temperature and pressure of food matrix and end up with cell destruction. This would increase the solvent to penetrate and dissolve the pectin into the extracting solvent resulting in the releasing of pectin to the solvent [22,40]. While UAE technique was generated the gaseous micro-bubbles during sonication resulting in the cavitation effect leading to cell disruption and larger pores on the surface of cell-matrix [4]. Moreover, Sucheta et al. [40] states that the UAE can disrupt the cell-matrix slower than microwave heating. 

Moreover, the MAE was synergized by UAE resulting in a higher pectin yield was found than only UAE or MAE was performed. This can be explained by the destruction of the cell walls of the raw materials during sonication, which would have allowed the microwave to easily transfer the compounds to the extracting medium. In addition, even though the UMAE conditions with the extraction time of MAE had been reduced twice, the pectin yield was comparable to the full extraction of MAE. Inversely, with the decrease of the UAE time, the pectin yield was found to be lower which could be pointed to the fact that the UAE had enhanced the pectin extraction efficacy in which sufficient UAE time (20 min) had been able to help in pectin extraction, while the MAE could also have helped to improve the pectin yields. 

Considering GalA content, it was found that the UAE pectin had shown the lower GalA content the MAE pectin, which could be explained by lower internal temperatures generated by cavitation effect which is lower by microwaves [40]. The GalA content of obtained pectin was positively related to pectin yield which the higher pectin yield was obtained, the higher GalA was found. This found was also in accordance with MAE results as well as the research conducted by Sucheta et al. [40] which was found in black carrot pectin. 

Moreover, the lycopene content in obtained pectin exhibited that MAE pectin contained higher trapped lycopene than UAE pectin. Moreover, the UMAE pectin which obtained from half UAE followed by MAE, had contained higher trapped lycopene than the UMAE pectin which was obtained from full-time UAE. Inversely to pectin yield, it was pointed that the UAE had negatively affected the trapped lycopene content in pectin which may be affected by the GalA content and also pectin molecule. However, the left the mixture of pectin and *n*-hexane for 24 h. could obviously release lycopene from the pectin and some maybe interact with pectin molecule via hydrophobic interaction. However, the structure analysis of obtained pectin could be more deeply studied. 

The fuzzy analytical method has been applied to evaluate the best extraction conditions by using three criteria, which were pectin yield, galacturonic acid, and lycopene content with weights of 75, 20, and 5, respectively, the same as those used for evaluating for UAE and MAE. The results indicated that the best UMAE conditions with UAE were followed by half of MAE. Therefore, pectin from these conditions were studied structure using FT-IR and compared to UAE pectin, MAE pectin, and commercial pectin as shown in Figure 2. It was found that the spectra of FTIR analysis confirmed the presence of functional groups in the fingerprint region (800–1200 cm^−1^) of identification for polysaccharide in all of the extracted pectin [41]. Besides, the peak in the range of 3200–3500 cm^−1^ which was attributed to the OH group of pectin molecule [41]. Moreover, it showed the peaks at 1416, 1712, and 1745 cm^−1^ which were pointed to the carboxylic group and carboxylic ester, respectively [42]. Moreover, the peak at 1201 cm^−1^ was found in the obtained pectin which was identified to be aliphatic carboxylic acid (and was probably citric acid). The total carboxyl group was calculated according to Liew et al. [27] by the accumulation of the absorbance intensity at 1745 cm^−1^ (methyl-esterified carboxyl groups) and at 1630 cm^−1^ (non-methyl-esterified carboxyl groups). The results showed that UMAE pectin was contained methyl-esterified carboxyl groups and non-methyl-esterified carboxyl groups comparable to UAE pectin and MAE pectin. This could be an indication that the extraction techniques were not affected by the main structure of the pectin.

## 4. Materials and Methods

### 4.1. Materials

The tomato wastes were obtained from Tomato Process Industry in Nong Khai Province, Thailand and were dried at 60 °C for 24 h. The ground dried tomato waste powder was kept in a plastic bag and stored at −18 °C prior to further experimentation.

### 4.2. Pectin Extraction

**Figure 3:**
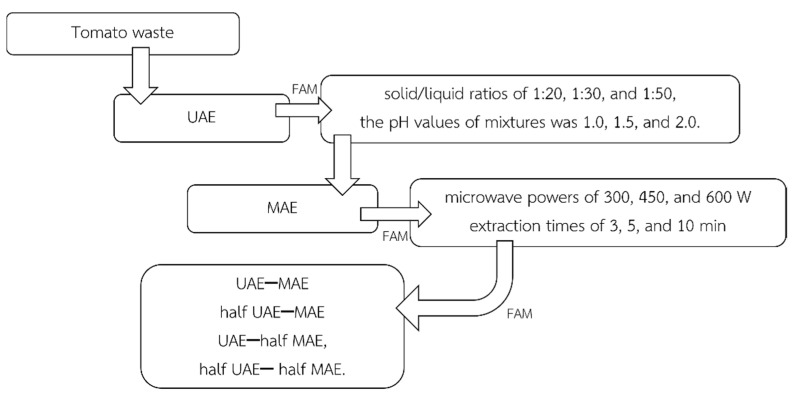
The schematic diagram of pectin extraction in this work.

Pectin from tomato waste powder was extracted with citric acid under different techniques and conditions. The schematic diagram is shown in Figure 3. 

Ultrasound-assisted extraction (UAE): The UAE was performed using an ultrasound bath with an ultrasonic frequency of 37 kHz. The UAE experiments were carried out according to a 3 × 3 full factorial design, the solid/liquid ratios (1:20, 1:30, and 1:50 g/mL) and the pH values (1.0, 1.5, and 2.0) were the independent values. The tomato waste powder was added to a citric acid solution at the different solid/liquid ratios and the pH values of mixtures. Moreover, different concentrations of citric acid were used (0.2 M, 0.30 M, 1 M, 1.35 M, and 2.5 M). The mixture was then sonicated at a temperature of 80 °C for 20 min in accordance with Sengkhamparn et al. [7].

Microwave-assisted extraction (MAE): The MAE was performed using a household microwave oven at a working frequency of 2450 MHz and the experiment was carried out regarding a 3 × 3 full factorial design with the micro-wave powers (300, 450, and 600 W) and at extraction times (3, 5, and 10 min) were the independent values. The tomato waste powder was added to citric acid solution (1.0 M) at the solid/liquid ratio of 1:20 and then the pH of the mixture was adjusted to 1.5. The temperature for each MAE condition was in the range of 70–100 °C. 

Ultrasound-microwave-assisted extraction (UMAE): To study the synergist effects of both techniques, the pectin from tomato waste powder was extracted by citric acid solution (1.0 M) at the solid/liquid ratio of 1:20 and a pH of 1.5 under the following conditions: (1) UAE, (2) MAE, (3) UAE–MAE, (4) half UAE–MAE, (5) UAE–half MAE, and (6) half UAE– half MAE. It was noted that the half conditions were performed twice under the reduction of extraction time. 

After extraction of each method, the mixture was cooled for 15 min and then centrifuged. The supernatant was poured into twice the volume of 95% (*v*/*v*) ethanol in order to precipitate the pectin and then was left for 24 h at 4 °C. The floating precipitant was discarded, while the remainder was rinsed twice with 70% (*v*/*v*) ethanol, and then dried at 60 °C for an hour using a vacuum oven before being placed in aluminum foil and kept in a desiccator for further analysis. The obtained pectin yield was calculated by comparing it to the weight of tomato waste powder.

### 4.3. Chemical Properties of Pectin

#### 4.3.1. Lycopene Contents

The lycopene contents were determined according to [7] with some modifications. Briefly, about 0.5 g of pectin was added to 5 mL of n-hexane and then left for 24 h. at room temperature in the dark. The lycopene content was then calculated according to [7] and was expressed as mg/100 g of pectin.

#### 4.3.2. Total Carboxyl Groups

The pectin obtained from the UAE method was determined using Fourier-transform infrared spectroscopy. The pectin samples were evaluated with wavelengths in the range of 4000–400 cm^−1^. The total carboxyl group was calculated according to Liew et al. [27] by the accumulation of the absorbance intensity at 1745 cm^−1^ (methyl-esterified carboxyl groups) and at 1630 cm^−1^ (non-methyl-esterified carboxyl groups). 

#### 4.3.3. Galacturonic Acid Content

The Galacturonic acid content of the MAE-pectin and the UMAE pectin was determined using m-hydroxydiphenyl and absorbance at 520 nm in which a standard solution of galacturonic acid (GalA) at concentrations of 20–100 μg/mL was compared. 

#### 4.3.4. Structural Analysis

The structure of pectin samples obtained from the UAE, MAE, and the UMAE methods was evaluated by using Fourier transform infrared spectroscopy (Perkin-Elmer, Spectrum One) with wavelengths ranging from 4000–400 cm^−1^.

### 4.4. Statistical Analysis

In the studies of the effects of MAE and UMAE, the data were recorded in triplicate and was then statically evaluated with full factorial design. In the study of the synergistic effects of both techniques, the data were also analyzed in triplicate and were statically evaluated with a completely randomized design. The treatment comparison was performed using Duncan’s New Multiple Range Test with a significance level of *p* < 0.05.

### 4.5. Fuzzy Analytical Method (FAM)

In order to determine the best pectin extraction conditions, FAM was applied for discission the best Microwave-assisted extraction conditions and also the best Ultrasound-Microwave-assisted extraction conditions according to the Lasunon [43]. The criteria for decision were the pectin yield, the galacturonic acid content, and lycopene content. The result of each criterion was calculated to score from 0 to 10 by using the lowest and highest value for each of the criteria. The overall performance index was calculated by using the weight of three criteria were the pectin yield with the weight of 75, the Galacturonic acid content with the weight of 20 and the lycopene content with the weight of 5. 

## 5. Conclusions

Pectin can be extracted by various techniques aiming to improve the extraction coefficient. The extraction pH values (0.1, 0.15 and 2.0) and the SL ratios (1:20, 1:30, and 1:50) of the UAE conditions at 80 °C and 20 min were studied. The results showed that the lower extraction pH and higher SL ratio did cause a higher pectin yield, but had resulted in a lower number of total carboxyl groups, as well as trapped lycopene content. For MAE technique for pectin extraction from the industrial tomato waste. The results showed that the microwave heating at high power gave the high pectin yield and high GalA content. Moreover, the combination of UAE to MAE could significantly synergist the pectin yield and also GalA content. However, the UMAE technique could reduce the harshness of individual technique while giving the higher pectin yield. The FT-IR spectra could point that the extraction techniques have no affected on the pectin structure from tomato waste. Interestingly, the obtained pectin showed the lycopene which may be trapped or interacted with pectin.

## Figures and Tables

**Table 1:** The pectin yield, galacturonic acid, and lycopene content of MAE pectin and overall performance index of each MAE condition.

MAE Conditions	Pectin Yield (%) ^1^	GalA Content (µg/L) ^1^	Lycopene Content (µg/g Pectin) ^1^	Overall Performance Index
300 W 3 min	9.43 ± 0.13 ^h^	24.91 ± 1.44 ^a^	27.89 ± 0.48 ^a^	2.50
300 W 5 min	15.10 ± 0.97 ^f^	19.01 ± 2.64 ^cd^	27.05 ± 0.65 ^bcd^	2.72
300 W 10 min	31.58 ± 0.48 ^a^	19.24 ± 1.67 ^cd^	26.74 ± 0.18 ^d^	7.23
450 W 3 min	12.57 ± 0.37 ^g^	17.56 ± 1.63 ^d^	27.57 ± 0.36 ^ab^	2.54
450 W 5 min	26.83 ± 0.46 ^d^	18.01 ± 1.25 ^d^	26.74 ± 0.36 ^d^	5.74
450 W 10 min	29.89 ± 1.75 ^b^	20.81 ± 2.50 ^bc^	26.74 ± 0.18 ^d^	6.83
600 W 3 min	22.85 ± 0.64 ^e^	20.54 ± 1.42 ^bc^	27.47 ± 0.31 ^abc^	5.70
600 W 5 min	29.67 ± 0.25 ^b^	14.91 ± 1.45 ^e^	26.85 ± 0.31 ^cd^	6.50
600 W 10 min	28.28 ± 0.56 ^c^	22.44 ± 0.47 ^b^	27.16 ± 0.00 ^bcd^	7.00

^1^ Different letter in each column shows the significant difference (*p* < 0.05) of the values.

**Table 2:** The pectin yield, galacturonic acid (GalA), and lycopene content of obtained pectin and overall performance index of each MAE condition.

Extraction Conditions	Pectin Yield (%) ^1^	GalA Content (µg/L) ^1^	Lycopene Content (µg/g Pectin) ^1^	Overall Performance Index
UAE	28.25 ± 2.14 ^bc^	13.41 ± 1.14 ^c^	9.99 ± 1.08 ^bc^	6.20
MAE	30.12 ± 0.12 ^b^	16.86 ± 1.08 ^b^	27.05 ± 0.18 ^a^	5.07
UAE + MAE	34.06 ± 1.11 ^a^	16.86 ± 0.76 ^b^	10.82 ± 0.18 ^b^	8.61
UAE + 1/2 MAE	34.39 ± 1.12 ^a^	19.59 ± 1.43 ^a^	8.53 ± 0.79 ^d^	8.99
1/2UAE + MAE	26.49 ± 0.18 ^c^	16.24 ± 2.47 ^b^	26.85 ± 0.00 ^a^	3.66
1/2UAE + 1/2 MAE	19.68 ± 1.61 ^d^	16.54 ± 0. 80 ^b^	9.26 ± 1.18 ^cd^	3.57

^1^ Different letter in each column shows the significant difference (*p* < 0.05) of the values.

## Data Availability

Data sharing is not applicable to this article.

## Sample Availability

Samples of the compounds are not available from the authors.
